# Interaction of Polygenetic Variants for Gestational Diabetes Mellitus Risk with Breastfeeding and Korean Balanced Diet to Influence Type 2 Diabetes Risk in Later Life in a Large Hospital-Based Cohort

**DOI:** 10.3390/jpm11111175

**Published:** 2021-11-10

**Authors:** Sunmin Park

**Affiliations:** Obesity/Diabetes Research Center, Department of Food and Nutrition, Institute of Basic Science, Hoseo University, YejunBio, 165 Sechul-Ri, BaeBang-Yup Asan-Si, ChungNam-Do, Asan 336-795, Korea; smpark@hoseo.edu; Tel.: +82-41-540-5345; Fax: +82-41-548-0670

**Keywords:** gestational diabetes mellitus, type 2 diabetes, genetic variants, Korean balanced diet, beast-feeding, prepregnant age

## Abstract

The etiologies of gestational diabetes mellitus (GDM) and type 2 diabetes mellitus (T2DM) are similar. Genetic and environmental factors interact to influence the risk of both types of diabetes. We aimed to determine if the polygenetic risk scores (PRS) for GDM risk interacted with lifestyles to influence type 2 diabetes risk in women aged >40 years in a large hospital-based city cohort. The participants with GDM diagnosis without T2DM before pregnancy were considered the case group (*n* = 384) and those without GDM and T2DM as the control (*n* = 33,956) to explore GDM-related genetic variants. The participants with T2DM were the case (*n* = 2550), and the control (*n* = 33,956) was the same as GDM genetic analysis for the interaction analysis of GDM genetic risk with lifestyles to influence T2DM risk. The genetic variants for the GDM risk were selected from a genome-wide association study (GWAS), and their PRS from the best model with gene-gene interactions were generated. GDM was positively associated with age at first pregnancy, body mass index (BMI) at age 20, and education level. A previous GDM diagnosis increased the likelihood of elevated fasting serum glucose concentrations and HbA1c contents by 8.42 and 9.23 times in middle-aged and older women. However, it was not associated with the risk of any other metabolic syndrome components. Breast-feeding (≥1 year) was inversely associated with the T2DM risk in later life. In the genetic variant-genetic variant interaction, the best model with 5-SNPs included *PTPRD*_rs916855529, *GPC6*_rs9589710, *CDKAL1*_rs7754840, *PRKAG2*_rs11975504, and *PTPRM*_rs80164908. The PRS calculated from the 5-SNP model was positively associated with the GDM risk by 3.259 (2.17–4.89) times after adjusting GDM-related covariates. The GDM experience interacted with PRS for the T2DM risk. Only in non-GDM women PRS was positively associated with T2DM risk by 1.36-times. However, long breastfeeding did not interact with the PRS for T2DM risk. Among dietary patterns, only a Korean-style balanced diet (KBD) showed an interaction with PRS for the T2DM risk. Participants with a low-PRS had the lowest serum glucose concentrations in the high KBD intake but not low KBD intake. In conclusion, participants with a high PRS for GDM risk are positively associated with T2DM risk, and breastfeeding for ≥1 year and consuming KBD offset the PRS for GDM risk to influence T2DM risk in middle-aged and older.

## 1. Introduction

Glucose homeostasis is a net of insulin resistance and insulin secretion. Type 2 diabetes (T2DM) is developed when their balance has deteriorated, i.e., insulin secretion cannot overcome increased insulin resistance, resulting in T2DM. Because Asians have lower insulin secretion capacity, they are susceptible to T2DM under increased insulin resistance states, such as aging and elevated inflammation. During the second trimester of pregnancy, insulin resistance increases to make glucose more available to the fetus. Pregnant women develop gestational diabetes mellitus (GDM) when they cannot overcome elevated insulin resistance by increasing insulin secretion. After the modernization of lifestyles in Asians, the prevalence of GDM is much higher in Asians than in Caucasians. In the 2007 Los Angeles Mommy and Baby Study, the GDM prevalence was 15.5% in Asian American women, 10.7% in Hispanic women, 9.0% in non-Hispanic black women, and 7.9% in non-Hispanic white women [1]. The GDM risk is 2.44 times higher than other ethnicities independent of the maternal age, marital status, prenatal care system, income, education, prepregnant body mass index (BMI), and physical exercise [1]. A higher GDM risk in Asians is associated with low insulin secretion capacity and β-cell mass than Caucasians.

As GDM has a similar etiology to T2DM, women with GDM have a higher risk of T2DM in later life when insulin resistance increases. The GDM risk is also similar to T2DM. The well-known GDM risk factors are maternal age, prepregnant BMI, weight gain, preterm delivery history, family history of diabetes, and inflammation status during the first trimester of pregnancy, indicating increased insulin resistance [2,3,4]. Furthermore, the maternal serum 25-hydroxy-vitamin D, magnesium, lysine, valine, tyrosine concentrations, and persistent organic pollutants, which are determined by aryl hydrocarbon receptor transactivating activity, act as GDM risk factors [5,6,7]. Most modifiable risk factors are involved in insulin resistance during pregnancy, and they can be modified with lifestyle changes, such as lower energy intake, saturated fat, and exercise. Women with GDM have a higher susceptibility to T2DM within several years after delivery, but few studies have revealed its risk in middle-aged adults [8].

**Figure 1:**
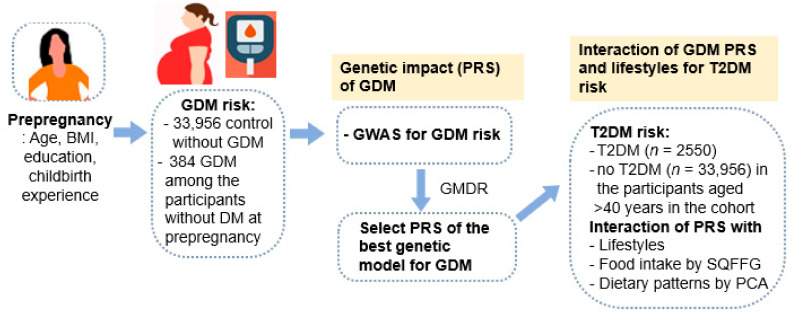
Research scheme of the present study. GDM, Gestational Diabetes Mellitus; GWAS, Genome-Wide Association Study; PRS, Polygenetic Risk Scores; GMDR, Generalized Multifactor Dimensionality Reduction; T2DM, Type 2 Diabetes Mellitus; DM, Diabetes Mellitus; SQFFQ, Semi-Quantitative Food Frequency Questionnaires; PAC, Principal Component Analysis.

The genetic variants influence insulin secretion and insulin resistance to affect the T2DM risk. The major genetic variants for the T2DM risk in Asians and Europeans are related to the β-function, even though some peroxisome proliferator-activated receptor-gamma (PPARγ), PPARγ coactivator 1-alpha (*PGC−1a*), and insulin receptor substrate−1 *(IRS1)* genetic variants are associated with the insulin sensitivity [9,10,11]. Potassium Voltage-Gated Channel Subfamily Q Member 1 (*KCNQ1*)_rs163177, CDK5 regulatory subunit associated protein 1, such as 1 (*CDKAL1*)_rs10946398 and rs7754840, insulin-degrading Enzyme (*IDE*)_rs4646957, ATP binding cassette transporter 1 (*ABCA1*)_rs4149313, and hematopoietically-expressed homeobox protein (*HHEX*)_rs5015480, which are mainly involved in insulin secretion and β-cell survival, are reported to be associated with T2DM [11,12,13]. They are inversely associated with the HOMA-B and area under the curve of serum insulin concentrations during the 100 g oral glucose tolerance test (OGTT) [10]. These results suggest that adults with the risk alleles of these genetic variants might reduce the insulin secretion capacity. Some genetic variants interact with lifestyle and nutrient intake [11]. High energy and alcohol intake interact with *CDKAL1*, *HHEX*, and *PGC−1a* genetic variants for the type 2 diabetic risk [11,12]. Common genetic variants of T2DM have been reported to be related to the GDM risk [14,15]. GDM-related genetic variants are related mainly to the β-cell function, such as *CDKAL1*_rs7754840, melatonin receptor 1B (*MTNR1B*)_rs10830963 and rs1387153, cyclin-dependent kinase inhibitor 2A/B (*CDKN2A/B*)_rs7020996, and *HHEX*_rs7923837 [16,17,18]. On the other hand, the genetic variants associated with adipogenesis and inflammation to increase insulin resistance, including fat mass and obesity-associated (*FTO*)_rs1421085, leptin receptor (*LEPR*)_rs1137100, and *PPARγ*_rs1801282, are also reported [19,20]. On the other hand, a few studies reported that women with a GDM risk had a higher risk of T2DM in middle age and older. We examined the hypothesis that the polygenetic risk scores (PRS) for GDM risk interacted with lifestyles to influence type 2 diabetes risk in middle-aged and elderly persons. This hypothesis was determined by exploring the PRS for GDM risk and the interaction of the PRS with lifestyles to influence T2DM risk in 36,890 women aged >40 years in a large hospital-based city cohort. The research scheme of the present study is presented in Figure 1.

## 2. Methods

### 2.1. Participants

This study was conducted with 36,902 women aged >40 years who participated in a hospital-based city cohort study of the Korean Genome and Epidemiology Study (KoGES), organized by the Korean Center for Disease and Control for ten years from 2004 to 2013. The institutional review boards of the Korean National Institute of Health (KBP-2015-055) and Hoseo University (1041231-150811-HR-034-01) approved the present study. All participants provided written informed consent.

### 2.2. Definition of GDM and T2DM

Those who answered “yes” for GDM diagnosis from the physician during pregnancy (*n* = 396) were considered the GDM case group, but those who had diabetes before pregnancy (*n* = 12) were removed from the GDM case group. Thus, the participants in the GDM case group initially diagnosed GDM during the pregnancy (*n* = 384). Type 2 diabetes (T2DM) was defined as fasting serum glucose concentrations >126 mg/dL, 6.5% HbA1c contents, or taking hypoglycemic agents when the women aged >40 years enrolled in the KoGES study. The T2DM case group included 2550 women currently having T2DM. The participants in the control group had neither experience of GDM nor T2DM until enrolling in the city hospital-based study (*n* = 33,956).

### 2.3. General Characteristics and Anthropometric and Biochemical Measurements

During a health interview, the participants provided age, gender, education, income, body weight at age 20, smoking status, alcohol consumption, and physical activity [21]. Menarche and menopause age were defined as initial menstruation and no menstruation for the last 12 months, respectively. They reported their experience of hormone replacement therapy, pregnancy, breastfeeding, and children. The education level was categorized into less than high school, high school, and college or more. The household income (USD/month) was low (<$2000), intermediate ($2000–4000), and high (>$4000) [22]. The smoking status was divided into current smokers, past smokers, and never-smokers [22]. The alcohol consumption status was classified into three categories according to the average daily alcohol consumption: nondrinker (0 g), mild drinker (0–20 g), and moderate drinker (>20 g) [22]. When the participants conducted moderate-intensity exercise for 30 min or heavy intensity exercise for 20 min more than three times a week (>150 min/week) for the last 3 months, they were categorized into regular physical exercise [23]. Moderate-intensity exercise included fast walking, mowing, badminton, swimming, tennis, and jogging. Basketball, soccer, and volleyball belonged to heavy intensity exercise [23]. The participants were divided into two groups with and without regular exercise.

The body weight, height, and waist circumference were measured using a standardized procedure [24]. The BMI was calculated by dividing the weight in kilograms by the height in meters squared. Overnight fasting blood was collected, and the plasma and serum samples were used for biochemical analysis [24]. The fasting plasma glucose and blood hemoglobin A1c (HbA1c; glycated hemoglobin) concentrations were determined using a Hitachi 7600 Automatic Analyzer (Hitachi, Tokyo, Japan). The blood pressure was determined at heart level in a sitting position using a sphygmomanometer.

### 2.4. Food Intakes Using a Semi-Quantitative Food Frequency Questionnaire (SQFFQ) and Dietary Pattern Analysis

During the previous year, the usual food intake was estimated with an SQFFQ designed and validated by KoGES [25]. The questionnaire included the frequencies and amounts of 106 food items, and the participants checked the intake of each food item. The daily intake of 106 food items was calculated by multiplying the frequencies of each food item by the assigned portion size over the previous year. The intake of 23 nutrients from the SQFFQ results was calculated using the Computer-Aided Nutritional Analysis Program (CAN Pro 3.0), a nutrient database prepared by the committee of the Korean Nutrition Society [25].

The intake of 106 food items from SQFFQ was categorized into 29 predefined food groups to determine dietary patterns using principal component analysis (PCA) under the FACTOR procedure. The 29 predefined food groups in the SQFFQ were included as independent variables in the PCA (Supplemental Table S1). The number of principal components was determined using the eigenvalues >1.5, and participants’ food intake was categorized into distinct dietary patterns. The orthogonal rotation procedure (varimax) was applied to the PCA, and factor-loading values ≥0.40 were considered to make significant contributions. PCA results met the criteria in 3 dietary patterns named as Korean-style balanced diet (KBD), Western-style diet (WSD), and rice-main diet (RMD) (Supplemental Table S2) [26]. The participants were divided into 2 groups according to the 70th percentiles of each dietary pattern [23,27]. The participants ≥70th percentiles of KBD indicated that they had a high KBD intake.

### 2.5. Genotyping DNA and Its Quality Control

The genomic DNA of each participant was extracted from whole blood, and their genotypes were assessed on an Affymetrix Korean Chip (Affymetrix, Santa Clara, CA), including the prevalent disease-related single nucleotide polymorphisms (SNPs) in Asians [28]. Biobank for Genome Science of the Korea National Institute of Health provided the genotype data. The genotyping accuracies of each DNA sample were examined by Bayesian Robust Linear Modeling using the Mahalanobis Distance Genotyping Algorithm [29]. After checking this algorithm, selected SNPs satisfied the following conditions: no gender bias, a genotyping accuracy of ≥98%, heterozygosity of <30%, a missing genotype call rate of <4%, and Hardy-Weinberg equilibrium (HWE) of *p* > 0.05 [26].

### 2.6. Best Models for Genetic Variant-Genetic Variant Interactions by Generalized Multifactor Dimensionality Reduction (GMDR)

**Figure 2:**
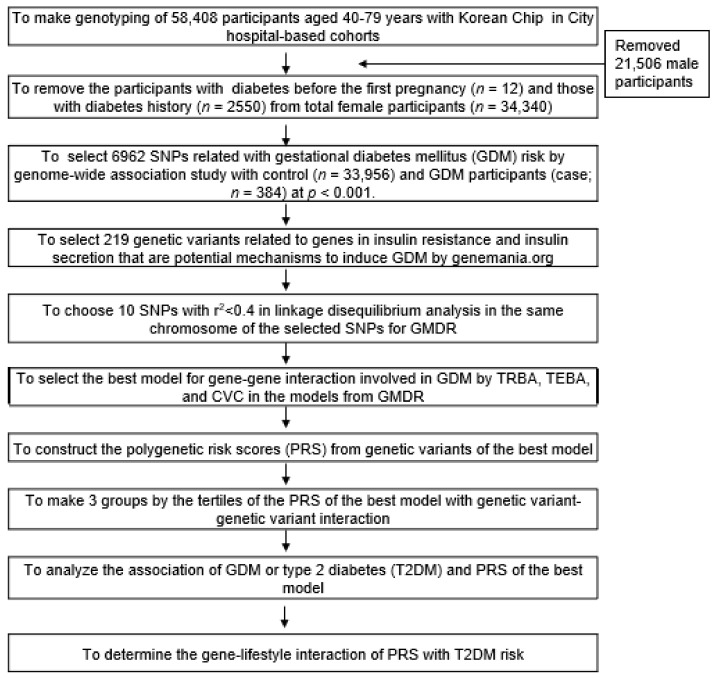
Flow chart to generate polygenetic variants in the best gene-gene interaction model for gestational diabetes mellitus (GDM) and their interaction with T2DM risk in later life. SNPs, single nucleotide polymorphisms; GMDR, Generalized Multifactor Dimensionality Reduction; TREB, trained balanced accuracy; TEBA, testing balanced accuracy; CVC, cross-validation consistency.

Figure 2 shows the process of selecting SNPs for GDM risk and making polygenetic risk scores (PRS). The genetic variants of the GDM risk were selected by conducting a genome-wide association study (GWAS) with GDM and control groups at *p*-values < 0.0001. The 6962 genetic variants were selected from the GWAS. The gene names corresponding to the selected genetic variants were determined using the g:Profiler website program (http://biit.cs.ut.ee/gprofiler/snpenses, accessed on 8 March 2021). The interaction of the genes corresponding to the selected genetic variants was checked using the Genmenia website (https://genmenia.org, accessed on 23 March 2021). Linkage disequilibrium (LD) analyses were conducted on the selected genetic variants in the identical chromosomes using Haploview 4.2 in PLINK to exclude the SNPs with strong LD (r^2^ > 0.3) that provided the same genetic variant information for the GDM risk. The genetic variants exhibiting strong LD were excluded from GMDR analysis. GMDR analysis was conducted to find the best mode with gene-gene interaction involved in glucose metabolism. The best model for gene-gene interactions to modulate the GDM risk was chosen with trained balanced accuracy (TRBA), testing balanced accuracy (TEBA), signed test between TRBA and TEBA, and cross-validation consistency (CVC) from the GMDR analysis. The genetic variants selected as the best model of SNP-SNP interactions were used to calculate the PRS models.

### 2.7. Statistical Analyses

Statistical analysis was performed using SAS version 9.3 (SAS Institute, Cary, NC, USA) and PLINK version 2.0 (http://pngu.mgh.harvard.edu/~purcell/plink, accessed on 2 March 2021). Using a GMDR program, the best gene-gene interaction model was selected using the *p*-values of <0.05 in the sign rank tests between TRBA and TEBA after adjusting for age at the first pregnancy, BMI at age 20 years, residence area, childbirth experience, and education level [30]. Ten-fold cross-validation was also used to check the CVC in the study with a >1000 sample size [30]. The non-risk and risk alleles of each SNP were counted as 0 and 1, respectively, to calculate the PRS of the best model with the SNP-SNP interaction from GMDR analysis [13]. For example, the TT, GT, and GG were given 0, 1, and 2, respectively, when the G allele was the risk allele of GDM. The PRS was calculated by adding the risk allele score of each SNP included in the best models. PRS obtained using the best model was divided into three categories: low-, medium-, and high-PRS, respectively. A high-PRS indicated the highest number of risk alleles in the best gene-gene-interaction model. The adjusted odds ratios (ORs) and 95% confidence intervals (CI) of the high-PRS were calculated for the GDM risk as a reference of the low-PRS in the PRS model after adjusting for the covariates. The covariates in model 1 included the age at first pregnancy, BMI at age 20 years, and residence area, while covariates in model 2 contained the covariates in model 1 plus childbirth experience and education level.

The descriptive statistics of categorical variables, such as gender and lifestyle, were calculated by the frequency distributions according to three PRS groups. The means and standard deviations were analyzed for the continuous variables according to GDM, T2DM, or PRS categories. The significance of the differences between the case and control groups of GDM and T2DM and three PRS groups was measured by one-way analysis of variance (ANOVA) after adjusting for the covariates. Multiple comparisons between the groups were conducted using a Tukey test. The frequency distributions of the classification variables were analyzed statistically using a chi-squared test.

GDM PRS interaction with each lifestyle (for example, breastfeeding) and nutrient intake was determined in two-way ANOVA with two main effects (GDM PRS and breastfeeding) and the interaction term of the main effects, after adjusting covariates to exclude the effects of the covariates. Each lifestyle parameter was dichotomized into the ‘high’ or ‘low’ groups according to the standardized or previously established values for the KoGES [12,23,27]. The association between GDM PRS and T2DM risk was separately determined in the low and high groups. Furthermore, fasting serum glucose concentrations and HbA1c contents were calculated according to GDM PRS in the low and high groups. These results indicated the interaction between GRS PRS and lifestyles to influence T2DM in later life. *p*-values of ≤0.05 were considered significant.

## 3. Results

General characteristics of the GDM and non-GDM: The participants with a GDM diagnosis were younger than those without a diagnosis. The participants with higher education and income had higher frequencies of GDM (Table 1). The age at first pregnancy and BMI at age 20 years were higher in the GDM group than in the non-GDM group. The BMI at age 20 years was positively associated with 7.6 times higher GDM risk than the non-GDM group. The frequency of macrosomia, GDM pregnancy outcomes, and mother age born macrosomia were much higher in the GDM group than in the non-GDM group. Macrosomia, an adverse outcome of GDM, was positively associated with a 2.09 times higher incidence than the non-GDM group (Table 1). There was no significant difference in the number of children and menarche age between the GDM and non-GDM groups.

Frequencies of breastfeeding and breastfeeding regardless of the periods were lower in the GDM group than in the non-GDM group (Table 1). Breastfeeding itself was not associated with the T2DM risk in middle-aged and elderly persons, but breastfeeding ≥1 year, but not <6 months, was inversely associated with the T2DM risk by 0.887 (0.806–0.976). GDM was positively associated with a 1.76 times higher MetS incidence risk in later life (Table 1). On the other hand, only the serum glucose concentrations and HbA1c contents at the fasting state were significantly higher in the GDM group than in the non-GDM group. GDM was positively associated with 8.42 and 9.23 times higher fasting serum glucose concentrations and HbA1c contents, respectively, in middle-aged and older adults than the non-GDM group (Table 1). The GDM increased the T2DM risk by 4.75 times in later life. On the other hand, the other components of MetS, such as waist circumferences, lipid profiles, and blood pressure, were similar in the GDM and non-GDM groups (Table 1). GDM was not significantly associated with the MetS components except for the serum glucose concentrations and HbA1c contents in middle-aged and older adults (Table 1).

### 3.1. Daily Nutrient Intake

According to the GDM and T2DM diagnosis, the nutrient intake did not show many differences among the groups. The energy intake was slightly higher in the non-GDM and no T2DM groups than in the other groups. In the GDM women, carbohydrate intake was lower in the participants without T2DM than those with T2DM, while fat intake showed an opposite trend. On the other hand, the protein, dietary fiber, and Na intake did not show significant differences among the GDM and T2DM groups. The vitamin C intake was higher in the participants without T2DM than in those with T2DM. The dietary patterns were categorized into a Korean balanced diet (KDB), Western-style diet (WSD), and rice-main diet (RMD). The incidence of T2DM was lower in the high intake of KBD, WSD, and RMD in the women without GDM, while women with a high WSD intake had a significantly lower T2DM incidence in the GDM group (Table 2). The percentage of smokers was significantly higher in the participants with T2DM in the non-GDM group. Drinking and exercise were similar among the GDM and T2DM groups.

### 3.2. Genetic Variants Associated with the GDM Risk and Its Best Model of Gene-Gene Interactions

Ten genetic variants were selected to influence GDM risk from the GWAS results, as listed in Table 3. Selected genetic variants belonged to the genes as follows: Protein phosphatase, Mg^2+^/Mn^2+^ dependent 1K *(PPM1K)_*rs6821589, fibroblast growth factor 2 (*FGF−2)_*rs189428800, CDK5 regulatory subunit associated protein 1, such as 1 *(CDKAL1)_*rs7754840, aryl hydrocarbon receptor *(AHR)_*rs181540079, AMP-activated protein kinase γ2 regulatory subunit *(PRKAG2)_*rs11975504, protein tyrosine phosphatase receptor type D *(PTPRD)_*rs916855529, interleukin 15 receptor subunit alpha *(IL15RA)_*rs2274034, protein phosphatase 1 regulatory subunit 12A *(PPP1R12A)_*rs148031082, glypican 6 *(GPC6)_*rs9589710, and protein tyrosine phosphatase receptor type M *(PTPRM)_*rs80164908 (Table 3). The genes were associated with insulin resistance and insulin secretion. On the other hand, unlike T2DM, genes were more likely to be related to insulin resistance than insulin secretion. *CDKAL1_*rs7754840 was positively associated with the T2DM risk in Korean adults.

The best model with SNP-SNP interaction from the selected 10 SNPs included 5 to 10 SNPs (Table 4). *PTPRD*_rs916855529, *GPC6*_rs9589710, *CDKAL1*_rs7754840, *PRKAG2* _rs11975504, and *PTPRM*_rs80164908 belonged to the 5 SNP model. The 6-SNP and 7-SNP models included *IL15RA*_rs2274034 and *AHR*_rs181540079 into the 5-SNP and 6-SNP models, respectively. Table 3 lists the other SNP models.

### 3.3. Association of the PRS with the 5-SNP Model with GDM and T2DM Risk

**Figure 3:**
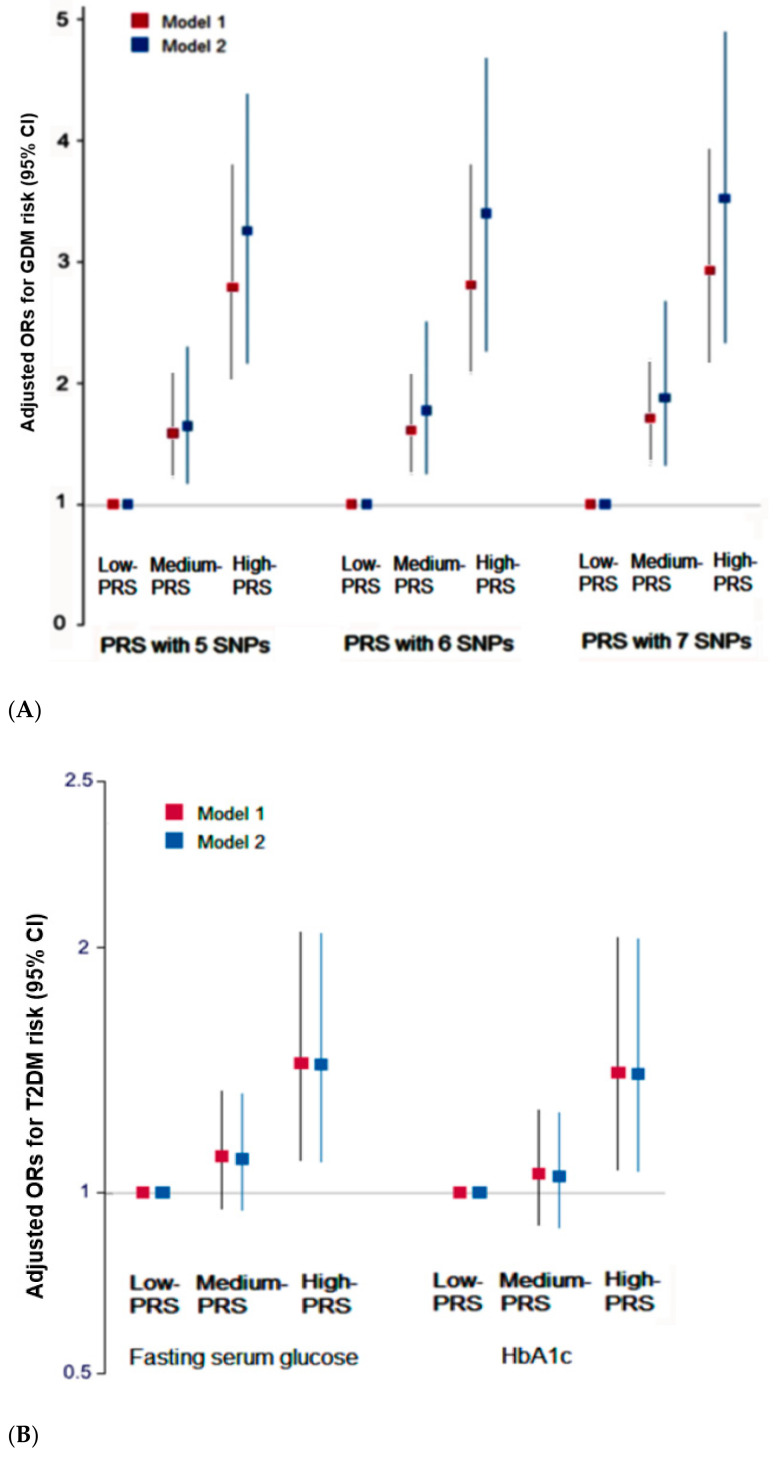
Adjusted odds ratio (ORs) for GDM or type 2 diabetes (T2DM) risk according to the groups of the low, medium, or high polygenetic risk scores (PRS) of the 5-, 6-, or 7-SNP models from GDM risk (**A**). Adjusted ORs and 95% confidence intervals (CI) for GDM according to the PRS groups of the 5-, 6-, or 7-SNP models from GDM risk. (**B**) Adjusted ORs and 95% confidence intervals (CI) for T2DM according to the PRS groups of the 5-, 6-, or 7-SNP models from GDM risk.

Figure 3 shows the adjusted ORs of PRS with the 5-SNP model for GDM, adjusting for covariates of age, BMI at age 20 years, residence area, education, and income (model 1) and the covariates in model 1 plus energy intake, alcohol, smoking, and exercise (model 2). The ORs and 95% CI of the PRS for GDM risk were similar in the 5-SNP, 6-SNP, and 7-SNP models in models 1 and 2 (Figure 3A). In PRS with the 5 SNP model, the adjusted ORs and 95% CI were 2.79 (2.05–3.80) in model 1 and 3.26 (2.17–4.89) in model 2 (Figure 3A). These results suggest that the participants with high-PRS had a 3.26 times more robust positive association with the GDM risk than the low-PRS group. Although the adjusted ORs were slightly elevated with increasing SNPs in the models, their increase was not high. The PRS groups of the 5-SNP model were used for the remaining analysis in the present study.

The PRS of the 5-SNP model for GDM risk was positively associated with the fasting serum glucose concentrations and HbA1c contents by 1.55 (1.15–2.08) and 1.48 (1.09–2.03) times, respectively (Figure 3B). On the other hand, the PRS was not associated with MetS and its components except for the serum glucose concentrations (Table S2). Thus, the PRS for GDM risk might influence the T2DM risk.

### 3.4. Interaction between PRS, GDM, and Dietary Patterns for T2DM Risk

**Figure 4:**
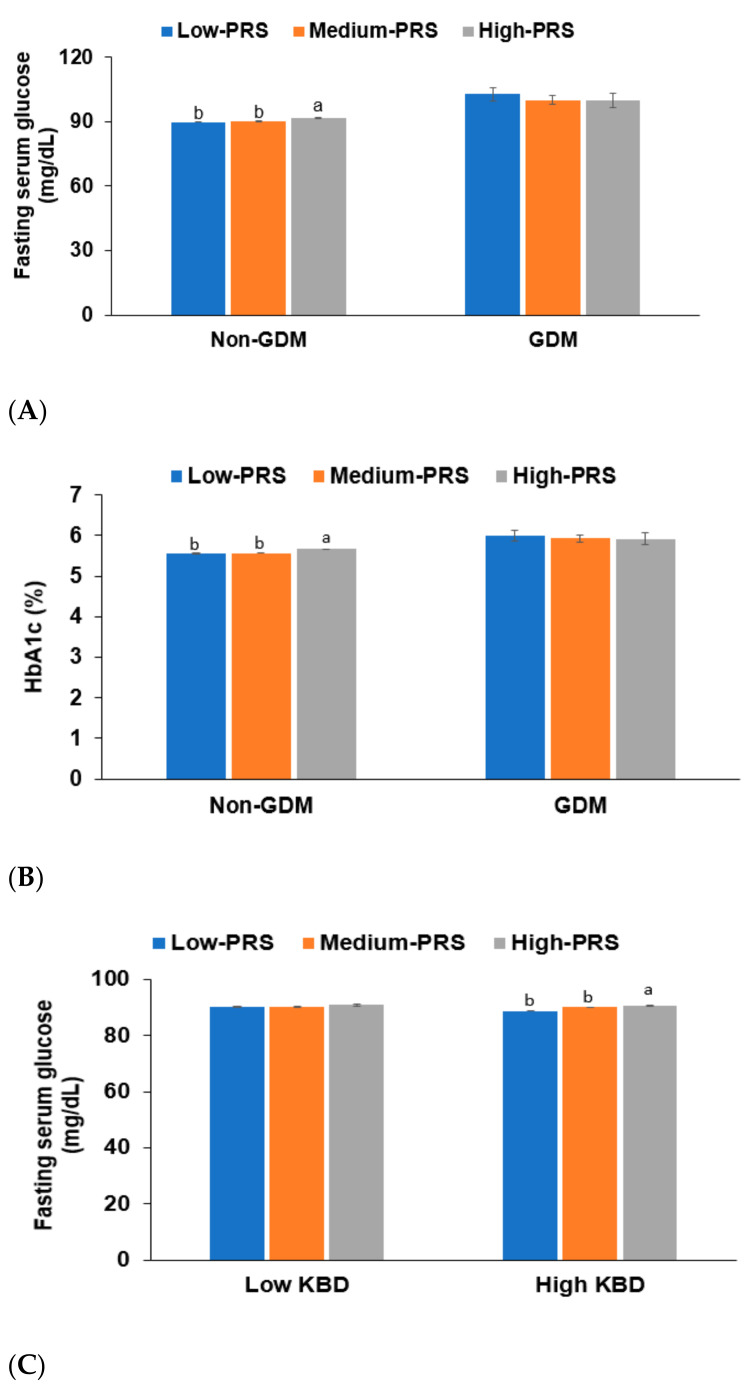
Adjusted means of the fasting serum glucose concentrations and HbA1c contents according to the groups of the low, medium, or high polygenetic risk scores (PRS) of the 5-SNP model. (**A**) Adjusted means and standard errors of fasting serum glucose concentrations in GDM and non-GDM groups according to the PRS groups of the 5-SNP models from GDM risk. (**B**) Adjusted means and standard errors of fasting serum glucose concentrations in GDM and non-GDM groups according to the PRS groups of the 5-SNP models from GDM risk. (**C**) Adjusted means and standard errors of fasting serum glucose concentrations in Low and High intake of KBD according to the PRS groups of the 5-SNP models from GDM risk.

The GDM diagnosis interacted with the PRS for the fasting serum glucose concentrations (*p* = 0.004) and HbA1c contents (*p* = 0.023) when the women became middle-aged and elderly (Table 5). PRS was positively associated with the risk of elevated fasting serum glucose concentrations and HbA1c contents only in participants with non-GDM. On the other hand, women with a GDM diagnosis elevated their concentrations to increase the T2DM risk regardless of PRS (Table 5). This result suggests that women with high PRS were susceptible to T2DM risk, and the genetic impact of the PRS was ignored to induce T2DM in later life when they were revealed GDM during the pregnancy. Women diagnosed with GDM were positively associated with the T2DM risk in women without a GDM diagnosis. On the other hand, breastfeeding periods did not interact with PRS to influence the T2DM risk. In the participants with non-GDM, the fasting serum glucose concentrations and HbA1c contents were higher in the High-PRS group than the Low-PRS group but not in the participants with GDM (Figure 4A). The HbA1c contents also showed similar patterns of the fasting serum glucose concentrations (Figure 4B). Among the three dietary patterns, PRS interacted only with KBD (*p* = 0.301; Table 5). There was a positive association between the PRS for T2DM risk in high KBD intake but not in a low KBD intake (Table 5). For participants with a high KBD intake, the fasting serum concentrations were lower in the Low-PRS group than the High-PRS group, but not for those with a low KBD intake (Figure 4C).

## 4. Discussion

GDM has a similar etiology to T2DM and is a well-known risk factor for T2DM. On the other hand, the genetic impact between GDM and T2DM was somewhat different in previous studies [11,12,16,31]. A few studies concluded that the genetic variants for GDM risk influenced the T2DM risk later in life. The present study investigated the hypothesis that the interaction of the PRS for GDM with dietary patterns affected the T2DM risk in 36,890 women aged >40 years of a large hospital-based city cohort. In the present study, the PRS for GDM risk in the best model with SNP-SNP interactions were *PTPRD*_rs916855529, *GPC6*_rs9589710, *CDKAL1*_rs7754840, *PRKAG2* _rs11975504, and *PTPRM*_rs80164908. Therefore, the PRS for GDM risk was positively associated with T2DM in middle-aged and older adults. Women with low-PRS lowered the glucose and HbA1C levels in the bloodstream in a high KBD intake. Long-term breastfeeding (≥1year) was inversely associated with T2DM, but it did not interact with the PRS for T2DM risk. It is novel to show the interaction of GDM PRS with breastfeeding and KBD to influence T2DM risk.

A GDM diagnosis is conducted at 24–28 weeks of pregnancy (second trimester) in Korea, mainly according to the two-step GDM diagnostic screening criteria. Pregnant women had a 50 g glucose challenge test at 24–28 weeks of gestation, and a 100 g glucose challenge was conducted if they had positive results (1-h glucose value ≥7.2 mM) [2,3]. The threshold values for the 100 g glucose load were 1 h (≥10.0 mM), 2 h (≥8.6 mM), and 3 h (≥7.8 mM) [2,8]. The GDM risk factors were multiparity ≥2, previous GDM diagnosis, stillbirth, abortion, preterm delivery, macrosomia, PCOS, high weight gain during the 1st trimester, age ≥ 25, BMI ≥ 25, and family history of diabetes [2,4], even though they showed inconsistent results. These risk factors are positively associated with GDM from 1.90 to 8.42-fold in the meta-analysis with 84 studies from 20 Asian countries [4]. The present study showed that women with ≥25 years, BMI at 20 years, and macrosomia experience increased the GDM risk by 1.170 and 7.60 fold, respectively, but the children’s number was not affected significantly. Therefore, women with maternal age >25 years and a high BMI need to be cautious about GDM during pregnancy.

The genetic variants for the GDM risk are somewhat different from those for T2DM, even though GDM and T2DM have a similar etiology. A meta-analysis showed that *MTNR1B* rs10830963 is positively associated with the GDM risk in Caucasians and Asians, but *TCF7L2* rs7903146 and *PPARG* rs1801282 were positively associated with GDM risk only in Asians [32]. In the Chinese population, some genetic variants related to obesity, such as *FTO*_rs1121980, *KCNQ1*_rs163182, *MC4R*_rs12970134, and *PROX1*_rs340841, were related to the GDM susceptibility [33]. The present study included the genetic variants for the GDM risk, such as *PPMIK*, *FGF2*, *AHR*, *PRKAG2*, *PTPRD*, *PPP1R12A*, and *PTPRM*, which were mostly involved in insulin sensitivity, but did not show high statistical significance with the T2DM risk in previous studies [10,12]. *GPC6* interacts with *PRKAG2*, *PTPRD*, *PTPRM*, and *CDKAL1*, shown in genemania.org, accessed on 23 March 2021, and their SNPs showed a gene-gene interaction in the present study. On the other hand, some genetic variants that influence insulin sensitivity and insulin secretion have been reported to be associated with GDM risk [34]. A recent study reported that AHR activation is associated with GDM risk and its genetic variant modulated its activation to modulate GDM risk [7]. T2DM-related genetic variants with the GDM risk are included in *CDKAL1*, *IGF2BP2*, *TCF7L2*, *KCNQ1*, and *MTNR1B* [34]. The present study also showed that *CDKAL1_*rs7754840 belonged to the polygenetic variants to influence the GDM risk. In Korean GDM women, *MTNR1B*_rs10830962 (OR = 1.454; *p* = 2.49 × 10^−13^) and *CDKAL1_*rs7754840 (OR = 1.518; *p* = 6.65 × 10^−16^) have a positive association with the GDM risk [17]. Therefore, the genetic traits in the GDM risk may be related to insulin sensitivity more than insulin secretion.

GDM is a critical risk factor for postpartum T2DM. Women with a GDM diagnosis develop T2DM within several years of pregnancy [8,35]. The GDM genetic impact on the postpartum T2DM risk is involved in environmental factors. The PRS of GDM risk was positively associated with an approximately 1.45 times higher T2DM risk in middle-aged and older women. A GDM experience interacted with the PRS to influence the T2DM risk. The non-GDM women were positively associated with 1.36 times higher T2DM risk. These results suggested that GDM women with a high-PRS had higher postpartum T2DM risk than those with a low-PRS. Rigorous glucose management during pregnancy prevents adverse infant outcomes, such as macrosomia, large for gestational age, and preterm delivery, but it does not protect against postpartum glucose disturbance, contributing to T2DM later [8]. On the other hand, breastfeeding is a protective factor for the postpartum T2DM risk in GDM women. The Diabetes & Women’s Health Study showed that longer lactation reduces the T2DM risk compared to 0 month (*p* trend = 0.003) while 12–24 months (HR = 0.85 [0.67–1.06]) and more than 24 months (0.73 [0.57–0.93]) of the lactation lowered the hazard ratio of T2DM [36]. These results are comparable to the present study that ≥1-year lactation in GDM women lowered the T2DM risk (OR = 0.887; 95% CI = 0.806–0.976). On the other hand, breastfeeding did not interact with the PRS of the GDM risk to influence T2DM in later life.

Lifestyles, including dietary patterns, nutrient intake, smoking, and drinking, can modulate the postpartum T2DM risk. KBD interacted with the PRS for GDM risk to influence T2DM risk in the present study. Only in women without GDM was the T2DM incidence lower in women with high KBD intake in the present study. The KBD included a diet high in beans, potatoes, kimchi, green vegetables, mushroom, white vegetables, seaweeds, fruit, and pickle. The KBD diet corresponds to high KHEI scores, a modified alternative healthy eating index (AHEI). A Mediterranean diet, DASH, and AHEI belong to mainly healthy food intake, including vegetables, legumes, fruits, poultry, and fish. A meta-analysis showed that they are inversely associated with T2DM risk (relative risk = 0.84; 95% CI = 0.77–0.91) [37]. Therefore, the KBD diet had protective activity against diabetes. Interestingly, the PRS for GDM risk interacted with KBD to influence T2DM later in the present diet. It suggests that women with high-PRS consume a KBD diet to reduce T2DM risk in later life.

This study had some limitations. This study was conducted by analyzing the data of a cross-sectional study. The results cannot be explained as a causal relationship. Second, GDM diagnosis was determined by a questionnaire of GDM diagnosis during the pregnancy from the doctor. The participants with a mean age of 50 recalled the body weight at 20 years and maternal age, including memory bias. However, self-reported prepregnancy weight has shown to be highly correlated with imputed prepregnancy weight (*r* = 0.98, *p* < 0.001, *κ* = 0.78) and their mean differences and their standard error are −1.7 and 0.1 kg in National Health and Nutrition Examination Survey 2003–2006, a representative sample of pregnant women in the USA [9]. It indicates that women have a substantial memory agreement about pregnancy. Third, we could not determine the effect of lifestyles, and food intake during prepregnancy and pregnancy on GDM risk since the questionnaires in KoGES did not include the lifestyles and food intake during pregnancy. The present study determined the interaction between the PRS for GDM risk and lifestyles to affect T2DM risk. Finally, usual food intake in the middle-aged and elderly participants was estimated using SQFFQ containing 106 common Korean foods used in KoGES during the last 1 year when the questionnaires were conducted. The SQFFQ was validated with the three-day food records in every 4 different seasons in a year under Korea Disease Control and Prevention Agency [23].

In conclusion, maternal age and prepregnant BMI were primary risk factors for GDM, while macrosomia and LGA were adverse outcomes of GDM. Breastfeeding ≥ 1 year was inversely associated with the T2DM risk in women with a GDM diagnosis. The PRS of the best model, including *PTPRD*_rs916855529, *GPC6*_rs9589710, *CDKAL1*_rs7754840, *PRKAG2*_rs11975504, and *PTPRM*_rs80164908, had a 3.26 times more robust positive association with the GDM risk and was 1.36 times more related to T2DM risk in middle-aged and older women. PRS for GDM risk interacted with serum glucose concentration and HbA1c contents, and only in non-GDM women PRS was positively associated with them by 1.36-times. The PRS for GDM risk had an interaction with the KBD intake for T2DM risk. Only in a high-KBD, was PRS of GDM risk positively associated with the T2DM risk, and the middle-aged and older adults with low-PRS had lower T2DM incidence than those with high-PRS. Therefore, women with a GDM risk recommended breastfeeding ≥1 year and maintaining KBD to reduce T2DM in middle-aged and older adults.

## Figures and Tables

**Table 1:** General characteristics of gestational diabetes mellitus (GDM) patients and adjusted odds ratio (OR) and 95% confidence intervals (CI) for GDM or type 2 diabetes (T2DM) in later life.

Parameters	Control ^1^ (*n* = 33,956)	GDM ^2^ (*n* = 384)	ORs and 95% CI for GDM ^3^
Age (<55 years) ^4^	53.0 ± 0.05	49.2 ± 0.45 ***	0.434 (0.310–0.607)
Education (Number, %)			
<high school	6773 (20.2)	44 (11.5) ***	1
High school	7678 (22.9)	55 (14.4)	0.915 (0.546–1.534)
≥College more	19,078 (56.9)	284 (74.1)	1.446 (1.017–2.381)
Income (Number, %)			
Low (<$2000)	3688 (11.0)	19 (5.1) ***	1
Medium ($2000–4000)	14,686 (43.8)	153 (40.0)	1.103 (0.675–1.803)
High (>$4000)	15,189 (45.3)	211 (54.9)	0.968 (0.586–1.600)
Age at first pregnancy (<25 years)	25.2 ± 0.02	26.4 ± 0.21 ***	1.170 (1.068 1.282)
BMI at age 20 (<25 kg/m^2^)	20.3 ± 0.08	22.2 ± 0.70 ***	7.600 (1.938–29.80)
Large baby (number, %)	326 (0.97)	55 (14.3) ***	2.085 (1.393–3.120)
Age born LGA (<29 years)	27.8 ± 0.11	29.1 ± 0.63 *	1.451 (0.544–3.868)
Children number (≤1)	161 (1.47)	223 (0.86)	0.830 (0.667–1.033)
Menarche age (<15 years)	15.0 ± 0.05	15.2 ± 0.47	0.838 (0.612–1.147)
Breast feeding (yes %)	31,088 (86.4)	301 (78.8) ***	
<1 year	25,218 (69.1)	212 (55.2) ***	0.920 (0.821–1.031) ^5^
≥1 year	16,292 (44.6)	104 (27.1) ***	0.887 (0.806–0.976) ^5^
Metabolic syndrome (*n*, %)	322 (1.01)	62 (1.22)	1.764 (1.279–2.434)
BMI (<25 kg/m^2^)	23.5 ± 0.02	23.5 ± 0.19	1.193 (0.879–1.620)
Waist circumference (cm) ^6^	78.2 ± 0.03	78.7 ± 0.25	1.345 (0.933–1.939)
Fasting serum glucose (<126 mg/dL)	90.9 ± 0.07	101 ± 0.66 ***	8.420 (6.452–10.99)
HbA1C (<6.5%)	5.59 ± 0.00	5.97 ± 0.03 ***	9.229 (6.368–13.38)
Type 2 diabetes (*n*, %)	2550 (7.6)	88 (22.9) ***	4.746 (3.314–6.796)
Serum total cholesterol (<230 mg/dL)	201 ± 0.20	199 ± 1.83	1.152 (0.886–1.498)
Serum LDL (<130 mg/dL)	119.2 ± 1.52	118.0 ± 11.2	1.076 (0.794–1.458)
Serum HDL (mg/dL) ^7^	56.4 ± 0.07	56.0 ± 0.67	1.083 (0.864–1.358)
Serum TG (<150 mg/dL)	112 ± 0.39	117 ± 3.68	1.163 (0.893–1.515)
SBP (<130 mmHg)	121 ± 0.08	121 ± 0.73	1.167 (0.913–1.491)
DBP (<90 mmHg)	74.3 ± 0.05	73.9 ± 0.44	1.221 (0.907–1.644)

^1^ The participants who answered “yes” for GDM experience but those who did not have T2DM before pregnancy were considered the case. ^2^ The participants who were not diagnosed with GDM and diabetes were considered as the control. ^3^ Adjusted means and ORs and 95% CI for GDM risk after adjusting the covariates, including age at pregnancy, BMI at 20 years, breastfeeding periods, residence area, childbirth experience, and education. ^4^ Adjusted ORs and 95% CI for T2DM risk. ^5^ (cutoff point values); ^6,7^ The cutoff points: ^6^ <90 cm waist circumferences (waist) for men <85 cm for women; ^7^ ≥40 mg/dL for men and ≥50 mg/dL serum high-density lipoprotein (HDL). LGA, large for gestational age; HbA1c, hemoglobin A1c; LDL, low-density lipoprotein; TG, triglyceride; SBP, systolic blood pressure; DBP, diastolic blood pressure. * significantly different from Control group at *p* < 0.05 and *** at *p* < 0.001.

**Table 2:** Nutrient intake of patients with gestational diabetes (GDM) diagnosis according to type 2 diabetes (T2DM).

Nutrients	Control ^1^		GDM ^2^	
	No T2DM (*n* = 33,956)	T2DM (*n* = 2550)	No T2DM (*n* = 296)	T2DM (*n* = 88)
Energy (EER %)	99.0 ^3^ ± 0.03 ^a^	98.5 ± 0.11 ^b^	98.3 ± 0.31 ^b^	98.1 ± 0.59 ^b#^
Carbohydrate (energy %)	72.0 ± 0.04 ^ab^	72.1 ± 0.14 ^ab^	71.2 ± 0.40 ^b^	72.9 ± 0.76 ^a^
Dietary fiber (g)	5.7 ± 0.01	5.6 ± 0.05	5.4 ± 0.13	5.8 ± 0.24
Fat (energy %)	13.7 ± 0.03 ^ab^	13.4 ± 0.11 ^b^	14.3 ± 0.3 ^a^	12.9 ± 0.57 ^b##^
Protein (energy %)	13.4 ± 0.01	13.4 ± 0.05	13.5 ± 0.15	13.4 ± 0.29
Sodium (mg/day)	2337 ± 6.31	2344 ± 23.5	2232 ± 65.9	2449 ± 126
Vitamin C (mg/day)	110 ± 0.32 ^a^	105 ± 1.22 ^b^	99.7 ± 3.42 ^b^	108.5 ± 6.54 ^ab+^
KBD (≥70th percentiles)	10,460 (29.7)	295 (22.7) ***	116 (34.0)	18 (41.9)
WSD (≥70th percentiles)	10,394 (29.5)	343 (26.3) *	135 (45.6)	28 (31.8) *
RMD (≥70th percentiles)	10,459 (29.7)	316 (24.3) ***	106 (31.1)	10 (23.3)
Smoking (current + past)	987 (2.91)	103 (3.97) ***	10 (3.39)	5 (5.69)
Drinking (g/day)	39.1 ± 1.27 ^a^	29.7 ± 3.83 ^b^	33.7 ± 10.7 ^b^	38.6 ± 20.5 ^a+^
Regular exercise (*n*, %)	17,722 (52.2)	1369 (53.7)	156 (52.7)	53 (60.2)

^1^ The participants who had “no” for GDM and T2DM diagnosis were considered the control. ^2^ The participants who answered “yes” for GDM diagnosis by a physician and those who did not have T2DM before pregnancy were considered the case. ^3^ Adjusted means after adjusting the covariates, including age, BMI at 20 years, current BMI, breastfeeding periods, alcohol intake, energy intake, residence area, income, education, exercise, and smoking. ^#^ Significantly different from T2DM by two-way ANOVA in continuous variables at *p* < 0.05 and ^##^ at *p* < 0.01. + Significant interaction between GDM and type 2 diabetes groups by two-way ANOVA at *p* < 0.05. ^a,b^ Different superscript letters indicated significant differences among the 4 groups in Tukey’s test at *p* < 0.05. * Significantly different from non-T2DM in categorical variables at *p* < 0.05 by χ2 test at *p* < 0.05 and *** at *p* < 0.001. KBD, Korean-style balanced diet; WSD, Western-style diet; RMD, rice-main diet.

**Table 3:** The characteristics of the genetic variants included in the genetic variant-genetic variant interaction analysis.

Chr ^1^	SNP ^2^	Position	Mi ^3^	Ma ^4^	OR and 95% CI ^5^	*p*-Value Adjusted ^6^	MAF ^7^	*p*-Value for HWE ^8^	Gene	Functional Consequence
4	rs6821589	89192792	G	A	2.86 (1.80–4.54)	7.84 × 10^−7^	0.011	0.547	*PPM1K*	intron
4	rs189428800	123779401	A	G	2.24 (1.45–3.45)	2.74 × 10^−5^	0.015	0.774	*FGF2*	intron
6	rs7754840	20661250	C	G	1.16 (1.00–1.35)	4.46 × 10^−5^	0.476	0.427	*CDKAL1*	intron
7	rs181540079	17370229	C	T	2.13 (1.37–3.31)	8.47 × 10^−5^	0.015	0.498	*AHR*	intron
7	rs11975504	151481965	C	T	1.73 (1.31–2.28)	1.04 × 10^−5^	0.051	0.553	*PRKAG2*	intron
9	rs916855529	8721355	G	A	0.73 (0.62–0.86)	1.28 × 10^−5^	0.397	0.160	*PTPRD*	intron
10	rs2274034	6019248	C	T	0.72 (0.08–0.62)	3.32 × 10^−6^	0.419	0.061	*IL15RA*	3′ UTR
12	rs148031082	80309656	A	G	2.48 (1.61–3.82)	3.86 × 10^−6^	0.014	0.129	*PPP1R12A*	intron
13	rs9589710	93967361	T	C	0.73 (0.62–0.86)	1.81 × 10^−5^	0.364	0.782	*GPC6*	intron
18	rs80164908	7862077	G	A	1.42 (1.18–1.71)	1.87 × 10^−5^	0.158	0.901	*PTPRM*	intron

^1^ Chromosome; ^2^ Single nucleotide polymorphism; ^3^ Minor allele; ^4^ Major allele; ^5^ Odds ratio (OR) and 95% confidence intervals (CI) for city hospital-based cohort; ^6^ *p*-value for OR after adjusting for age at first pregnancy, BMI at age 20, residence area, childbirth experience, education in the city cohort; ^7^ Minor allele frequency; ^8^ Hardy-Weinberg equilibrium. *PPM1K*, Protein phosphatase, Mg2+/Mn2+ dependent 1K; *FGF−2*, Fibroblast growth factor 2; *CDKAL1*, CDK5 regulatory subunit associated protein 1, such as 1; *AHR*, Aryl hydrocarbon receptor; *PRKAG2*, AMP-activated protein kinase r2 regulatory subunit; *PTPRD*, Protein tyrosine phosphatase receptor type D; *IL15RA*, Interleukin 15 receptor subunit alpha; *PPP1R12A*, Protein phosphatase 1 regulatory subunit 12A; *GPC6*, Glypican 6; *PTPRM*, Protein tyrosine phosphatase receptor type M.

**Table 4:** Generalized multifactor dimensionality reduction (GMDR) results of multi-locus interaction with genes related to gestational diabetes mellitus.

Model	Adjusted Age at First Pregnancy, and Weight at 20				Adjusted Age at First Pregnancy, Weight at 20, Residence Area, Childbirth Experience, Education			
	TRBA	TEBA	*p* Value	CVC	TRBA	TEBA	*p* Value	CVC
*PTPRD*_rs916855529	0.536	0.501	4 (0.828)	7/10	0.532	0.514	6 (0.377)	7/10
*GPC6*_ rs9589710 plus model 1	0.558	0.529	9 (0.010)	5/10	0.549	0.527	8 (0.055)	7/10
*CDKAL1*_rs7754840 plus model 2	0.581	0.550	8 (0.055)	8/10	0.568	0.536	8 (0.055)	7/10
*PRKAG2*_ rs11975504 plus model 3	0.601	0.543	9 (0.011)	5/10	0.587	0.547	9 (0.011)	9/10
*PTPRM*_rs80164908 plus model 4	0.624	0.555	10 (0.001)	10/10	0.608	0.547	9 (0.011)	10/10
*IL15RA_*rs2274034 plus model 5	0.644	0.548	10 (0.001)	9/10	0.626	0.546	10 (0.001)	9/10
*AHR*_rs181540079 plus model 6	0.663	0.557	10 (0.001)	10/10	0.643	0.549	10 (0.001)	10/10
*PPM1K*_ rs6821589 plus model 7	0.677	0.563	10 (0.001)	10/10	0.655	0.557	10 (0.001)	10/10
*PPP1R12A*_rs148031082 plus model 8	0.689	0.555	10 (0.001)	9/10	0.666	0.544	10 (0.001)	9/10
*FGF2*_rs189428800 plus model 9	0.699	0.553	10 (0.001)	10/10	0.674	0.544	10 (0.001)	10/10

TRBA, trained balanced accuracy; TEBA, test balance accuracy; CVC, cross-validation consistency; sign test, result, and *p*-value for the significance of GMDR model by sign test with and without adjusting for covariates designated in the table; BMI, body mass index.

**Table 5:** Adjusted odds ratios of polygenetic risk scores of the 5-SNP model (PRS) for type 2 diabetes (T2DM) risk after covariate adjustments according to the patterns of lifestyles and the interaction of PRS with lifestyles for T2DM.

	Low-PRS	Medium-PRS	High-PRS	Interaction
Non-GDM	1	1.009 (0.805–1.264)	1.362 (1.002–1.857)	0.0004 for FSB
GDM	1	1.429 (0.777–2.629)	1.115 (0.502–2.477)	
Non-GDM	1	1.005 (0.801–1.259)	1.358 (1.003–1.851)	0.023 for HbA1c
GDM	1	1.252 (0.545–2.874)	1.543 (0.567–4.203)	
Short period for BF (<1 year)	1	1.226 (0.925–1.625)	1.570 (1.049–2.349)	0.216
Long period for BF (≥1 year)	1	1.046 (0.776–1.411)	1.526 (0.991–2.351)	
Low intake of KBD (<70th percentile)	1	0.998 (0.705–1.412)	1.526 (0.934–2.494)	0.030
High intake of KBD (≥70th percentile)	1	1.117 (0.907–1.375)	1.503 (1.113–2.030)	
Low intake of WSD (<70th percentile)	1	1.129 (0.920–1.387)	1.623 (1.214–2.169)	0.193
High intake of WSD (≥70th percentile)	1	1.373 (0.950–1.986)	2.046 (1.240–3.377)	
Low intake of RMD (<70th percentile)	1	1.138 (0.927–1.398)	1.523 (1.128–2.055)	0.234
High intake of RMD (≥70th percentile)	1	1.537 (1.092–2.163)	2.048 (1.273–3.292)	

Values represent adjusted means and standard errors, adjusted odds ratio (ORs), and 95% confidence intervals (CI). Covariates included age, sex, education, income, energy intake (percentage of estimated energy requirement), job, residence areas, daily activity, alcohol intake, and smoking status. PRS with 5 SNPs of the best GMDR model was divided into three categories according to the number of the risk alleles: when the number of risk alleles in the PRS was ≤3, 4−5, and ≥6 into Low-PRS, Middle-PRS, and High-PRS, respectively. The reference for logistic regression was the low-PRS. Interaction between PRS and other factors was analyzed with multivariate regression models, including the corresponding main effects, gene interaction terms, and main effects and covariates. FSB, fasting serum glucose concentrations; HbA1c, hemoglobin A1c; GDM, gestational diabetes; BF, breastfeeding; KBD, Korean-style balanced diet; WSD, Western-style diet; RMD, rice-main diet.

## Data Availability

The author’ raw data involved in this study will be available to any qualified researcher.

## Supplementary Materials

The following are available online at https://www.mdpi.com/article/10.3390/jpm11111175/s1, Table S1: Factor loadings of food groups in dietary patterns identified using principal component analysis, Table S2: Adjusted means and odds ratios for the risk of metabolic syndrome and its components by polygenetic risk scores of the 5 SNPs model (PRS) for gene-gene interaction after covariate adjustments.
