# Supplementary material for: Interaction of Polygenetic Variants for Gestational Diabetes Mellitus Risk with Breastfeeding and Korean Balanced Diet to Influence Type 2 Diabetes Risk in Later Life in a Large Hospital-Based Cohort
Source: J Pers Med. 2021 Nov 10;11(11):1175. doi: 10.3390/jpm11111175 (PMC8619899; doi:10.3390/jpm11111175)
Supplement: Supplementary file 1 [file jpm-11-01175-s001.zip › jpm-1406649-supplementary.pdf]

**Table S1.** Factor loadings of food groups in dietary patterns identified using principal component analysis.

| | Factor1 | | Factor2 | | Factor3 | |
|---|---|---|---|---|---|---|
| Rice | -7 | | -6 | | 91 | * |
| Grain | 6 | | 5 | | -90 | * |
| Noodles | 1 | | 59 | * | 6 | |
| Breads | 9 | | 61 | * | -3 | |
| Cookies | 9 | | 34 | | 4 | |
| Beans | 54 | * | 6 | | -2 | |
| Potatoes | 47 | * | 13 | | -4 | |
| Kimchi | 42 | * | -4 | | -1 | |
| Eggs | 32 | | 20 | | 1 | |
| Fast foods | 1 | | 78 | * | 2 | |
| Green vegetables | 79 | * | 3 | | 2 | |
| Mushroom | 74 | * | 4 | | 6 | |
| White vegetables | 62 | * | 0 | | -2 | |
| Fatty fish | 55 | * | 15 | | 4 | |
| White fish | 62 | * | 15 | | 6 | |
| Crabs | 40 | * | 19 | | 8 | |
| Processed meats | 24 | | 6 | | -2 | |
| Red meats | 33 | | 37 | | 16 | |
| Soups | 10 | | 67 | * | 3 | |
| Chickens | 22 | | 37 | | 12 | |
| Seaweeds | 58 | * | 3 | | -4 | |
| Milk | 37 | | 7 | | -2 | |
| Beverages | 35 | | 6 | | -1 | |
| Coffee | -1 | | 11 | | 17 | |
| Tea | 4 | | 18 | | 17 | |
| Fruit | 42 | * | 1 | | -7 | |
| Pickle | 40 | * | 3 | | 6 | |
| Alcohol | 0 | | 6 | | 9 | |
| Nuts | 27 | | 11 | | -10 | |
| Variance Explained by Each Factor | 4.487 | | 2.381 | | 1.783 | |

Printed values are multiplied by 100 and rounded to the nearest integer. Values greater than 0.4 are flagged by an '*'.

**Table S2.** Adjusted means and odds ratios for the risk of metabolic syndrome and its components by polygenetic risk scores of the 5 SNPs model (PRS) for gene-gene interaction after covariate adjustments.

| | Low-PRS[1] (n-13,652) | Medium-PRS (n=16,679) | High-PRS (n=3,463) | Adjusted ORs (95% CI) [2] |
|---|---|---|---|---|
| Serum glucose[3] | 90.6±0.58 | 90.9±0.55 | 92.9±1.10 | 1.545 (1.148-2.079)<br>1.085 (0.874-1.348) |
| HbA1c[4] | 5.56±0.02 | 5.55±0.03 | 5.61±0.05 | 1.482 (1.085-2.025)<br>1.047 (0.835-1.313) |
| BMI [5] | 23.3±0.17 | 23.7±0.16 | 23.0±0.33 | 0.932 (0.845-1.028)<br>1.032 (0.973-1.095) |
| Waist (cm)[6] | 77.6±0.35 | 78.9±0.67 | 77.3±0.86 | 0.927 (0.826-1.041)<br>0.977 (0.911-1.048) |
| MetS[7] | 1379 (10.5) | 1799 (10.8) | 425 (11.0) | 1.075 (0.939-1.230)<br>1.083 (0.993-1.181) |
| Serum total cholesterol[8] | 203±1.60 | 201±3.05 | 200±3.90 | 0.991 (0.895-1.097)<br>0.973 (0.914-1.036) |
| Serum HDL[9] | 57.6±0.58 | 57.5±1.09 | 56.8±1.39 | 1.025 (0.937-1.121)<br>0.985 (0.932-1.040) |
| Serum LDL[10] | 121±1.47 | 119±2.80 | 116±3.59 | 0.981 (0.874-1.102)<br>0.994 (0.926-1.067) |
| Serum TG[11] | 121±3.80 | 124±7.26 | 137±9.29 | 1.017 (0.918-1.126)<br>0.978 (0.919-1.042) |
| SBP[12] | 118±0.60 | 119±1.13 | 119±1.46 | 0.996 (0.945-1.049)<br>0.965 (0.885-1.052) |
| DBP[13] | 73.1±0.53 | 73.2±0.50 | 73.1±1.01 | 0.908 (0.826-1.003)<br>0.979 (0.925-1.036) |

[1]PRS of 5-SNPs in the best model were calculated by summing the number of risk alleles of SNPs. The PRS was divided into three categories (0-3, 4-5, and ≥6).

[2]Adjusted means and odds ratio (ORs) and 95% confidence intervals (CI) after adjusting the covariates including age, BMI at 20 years, current BMI, breast feeding periods, alcohol intake, energy intake, residence area, income, education, exercise, and smoking.

The cutoff points were as following: [3]<126 ml/dL fasting serum glucose plus diabetic drug intake; [4]<6.5% hbA1c plus diabetic drug intake [5]< 25 kg/m2 body mass index (BMI); [6]<90 cm waist circumferences (waist) for men <85 cm for women; [7] metabolic syndrome (MetS) definition; [8]<230 mg/dL fasting serum total cholesterol; [9]≥40 mg/dL for men and ≥50 mg/dL serum HDL; [10]<160 mg/dl serum LDL; [11] <150 mg/dl serum triglyceride; [12]<130 mmHg SBP and [13]<90 mmHg DBP or taking hypotensive medication.
